# Within‐ and Across‐Generational Effects of Temperature: Exposure of *Manduca sexta* Larvae to Heat Stress Impacts Future Reproduction and Offspring Development

**DOI:** 10.1002/ece3.72303

**Published:** 2025-10-12

**Authors:** Meggan A. Alston, Joel G. Kingsolver, Christopher S. Willett

**Affiliations:** ^1^ Department of Biology University of North Carolina at Chapel Hill Chapel Hill North Carolina USA

**Keywords:** carry‐over effects, development time, fecundity, parental effects, plasticity, thermal stress

## Abstract

The effects of temperature on reproduction and other key fitness traits are often primarily considered only for the adult thermal environment, but exposure to thermal stress during earlier life stages may carry over to influence adult traits within a generation or even across generations. In this study, we assessed how an acute heat shock event experienced at two different points in *Manduca sexta* larval development (early and late) impacted adult performance and fitness traits and whether thermal exposure of parents elicited plastic changes in offspring traits. Heat stress during late larval development had significantly greater negative impacts on adult performance and fitness compared to earlier exposure. Adults that experienced a late larval heat shock failed to produce any viable offspring due to complete elimination of egg hatching success. Larval heat stress during the parental generation also reduced larval development times of their offspring in both control and heat shock conditions. The results of this study illustrate the negative consequences of larval heat stress for adult fitness and indicate that the parental early thermal environment can significantly influence some traits in the next generation. The effects of parent environmental conditions during development, and not just at the adult stage, may therefore be an important but often overlooked factor when assessing cumulative fitness impacts across generations and predicting the vulnerability of populations to climate change.

## Graphical abstract

We assessed how an acute heat shock event experienced at two different points in *Manduca sexta* larval development (early and late) impacted adult performance and fitness traits and whether thermal exposure of parents elicited plastic changes in offspring traits. Differences in the timing of heat shocks in the larval period lead to dramatically different impacts on adult reproduction (with complete sterility for later larval stage heat shocks). Heat exposure had impacts in the next generation as well, leading to differences in developmental rates for offspring of heat‐shocked parents.

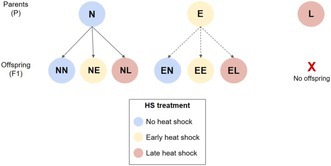

## 1 Introduction

Performance and fitness of organisms vary with environmental conditions, particularly with the thermal environment. For ectotherms, temperature strongly influences physiological rate processes (feeding and metabolic rate), life history traits (age, size at maturity), and fitness (survival, reproduction) (Angilletta 2009; Clarke 2017; Harrison et al. 2012). Therefore, exposure to temperatures outside of optimal thermal limits can have significant negative consequences for both individuals and populations. As temperatures vary across daily, seasonal, and annual time scales, individuals from different life stages and seasonal generations can experience differences in magnitudes and patterns of thermal exposure (Kingsolver et al. 2011). While it is well documented that thermal sensitivity often differs among life stages (Banahene et al. 2018; Barnes et al. 2019; Cavieres et al. 2020; Klockmann et al. 2017; Lockwood et al. 2018; MacLean et al. 2016; Petersen et al. 2000; Wang et al. 2004; York and Oberhauser 2002), the extent to which thermal exposure at one stage may result in lasting impacts across life stages or even subsequent generations has received much less attention.

Conditions experienced during earlier developmental (e.g., egg, larval, nymph, pupal) stages may affect physiology and development beyond the metamorphic boundary, resulting in impacts on adult traits (O'Connor et al. 2014; Pechenik 2006; Tejedo et al. 2010). Such examples have been demonstrated in multiple insect species, where exposure to high, sub‐lethal temperatures at pre‐adult stages results in negative fitness and performance consequences for adults, including impaired development and morphology, and reduced longevity and fecundity (Arbogast 1981; Green et al. 2019; Saxena et al. 1992). However, there is considerable variation across studies in the degree to which thermal stress during earlier development impacts later stages. Specific treatment conditions such as the intensity or timing of heat stress events may contribute to the variation in reported responses. Studies testing acute, short‐term heat stress during early development in insects have not found long‐lasting effects on female fecundity and other adult phenotypes (Knapp and Nedvěd 2013; Ma et al. 2004; Zhang, Chang, et al. 2015), but a study in *Plutella xylostella* did find that chronic (moderate, longer‐term) stress at early stages induced negative carry‐over effects on adult performance (Zhang, Rudolf, and Ma 2015). Heat shocks of *Manduca sexta* eggs reduced early larval growth but did not have lasting effects on later larval or adult stages (Potter et al. 2011). However, for some insect species, heat stress later in larval development may have more detrimental impacts on adult reproduction than earlier stages (Ma et al. 2004; Zhang, Chang, et al. 2015).

The thermal sensitivity of an individual at a given temperature may also be altered by prior thermal exposure (i.e., thermal plasticity). Many empirical studies in insects have documented positive effects of thermal plasticity (acclimation), such as increased thermal tolerance and improved survival during subsequent temperature stress (Chidawanyika et al. 2017; Huang et al. 2007; Li et al. 2018). However, plasticity in thermal response may not always be beneficial. For example, temperatures that facilitate acclimation for short durations of exposure can have negative impacts over longer periods or if repeated. The impact of thermal history on an organism's response therefore depends on interactions between timing, duration, and magnitude of past temperature exposures, i.e., time‐dependent effects (Bozinovic et al. 2011; Folguera et al. 2011; Kingsolver et al. 2015, 2020). Capacity for thermal plasticity also may differ across life stages (Carter and Sheldon 2020). Additionally, while thermal plasticity may benefit some traits (e.g., improved survival and growth rates), it could simultaneously result in trade‐offs for other traits, such as reduced reproduction (Cavieres et al. 2020; Koussoroplis et al. 2017).

Most studies of thermal plasticity have focused on within‐generation effects, but thermal exposure across generations can also have measurable impacts for organisms (Cavieres et al. 2019, 2020). Parents may be able to alter the phenotype of their offspring through a range of non‐genetic or epigenetic processes. These include maternal effects, such as offspring provisioning and care (Mousseau and Fox 1998), as well as epigenetic transmission (e.g., RNA‐mediated modifications, epigenetic marks, and DNA methylation) (Heard and Martienssen 2014; Ho and Burggren 2010). These processes can be altered by the environment, providing a potential mechanism by which the parental environment can influence the performance of offspring. The magnitude of across‐generational effects (aka parental effects) can be as strong as within‐generation effects for some traits, as documented by one study in a freshwater snail (Leicht and Seppälä 2019). Another study in *Daphnia* found that parental effects can persist for multiple generations after an initial thermal exposure (Walsh et al. 2014). Similar to plasticity within a generation, thermal plasticity carried through to the next generations can have either positive or negative impacts on offspring performance and fitness. Negative parental effects have been shown to decrease offspring survival and reproduction in *Tribolium* (Sales et al. 2018) and reduce thermal tolerance in *Caenorhabditis* (Sikkink et al. 2014). However, a study in *Drosophila* found evidence of improved offspring thermal tolerance after parental thermal stress exposure (Green et al. 2019). While most studies have focused on maternal effects (Massamba‐N'Siala et al. 2014; Zizzari and Ellers 2014), paternal effects could be important as well and may be more likely to result in negative consequences to offspring compared with maternal effects (Guillaume et al. 2016; Sales et al. 2018). Despite increasing evidence that considering across‐ in addition to within‐generation plasticity is likely important for shaping thermal performance, little is known about the specific conditions that may facilitate these effects. Most studies have only considered the adult parental environment or have evaluated thermal conditions applied across the entirety of a parent's lifetime. However, the particular timing, magnitude, and length of cues required to produce parental effects, especially during development (i.e., juvenile environmental conditions of parents), have not been well evaluated (Donelson et al. 2016, 2018; Schulte et al. 2011).

In this study, we explored the consequences of thermal exposure at different stages of larval development for adult performance and fitness traits (within a generation) and also tested for across‐generational effects of parent thermal exposure for offspring performance and fitness. We did this using a model insect system, *Manduca sexta* , to address two specific questions: (1) How does the timing of sub‐lethal heat stress, either at early or late larval stages, alter adult reproductive traits, and (2) Can thermal stress experienced during these distinct larval stages by parents elicit plastic changes in their offspring in similar thermal environments?

Previous experimental studies using *M. sexta* have thoroughly documented the effects of constant, fluctuating, and extreme temperatures for larval growth, development, survival and tolerance (Casey 1977; Kingsolver et al. 2009, 2015, 2016, 2020; Kingsolver and Woods 1997, 2016; Reynolds and Nottingham 1985). Heat stress of eggs can affect early larval development (Potter et al. 2011), but the consequences of heat stress of larvae on adult performance and fitness—and for the next generation—have not been evaluated. Based on prior studies, we predicted that early and late larval heat stress may differ in magnitude and direction of effects across traits (e.g., adult mass and development time, survival, and reproduction). *M. sexta* larval stages show differences in thermal sensitivity; heat waves experienced at 3rd—4th instar larval stages had the largest negative effects on survival to pupation compared with 1st, 2nd and 5th instars and resulted in longer development times and smaller mass gain during the final larval stage (Kingsolver et al. 2021). Heat shocks later during larval development may be more likely to reduce adult fecundity and result in across‐generational effects. In *M. sexta* , the final instar stage (5th) is a critical time for mass gain (Reinecke et al. 1980), early reproductive system development (Reinecke et al. 1983), and imaginal disk formation (Rosero et al. 2019). Imaginal disk are important regulators of developmental timing in holometabolous insects (Stieper et al. 2008); given that they develop into adult organs, they could be relevant for passing on signals about environmental conditions from larval to adult stages. To test for potential differences in responses between larval stages, in the current study we chose to focus on heat stress at early (3rd) and late (5th) instars to determine the fitness impacts on both adults and the next generation.

## 2 Methods

### 2.1 Study System

The tobacco hornworm, *Manduca sexta* (Lepidoptera: Sphingidae), is a well‐established model system for the study of insect development, physiology, and thermal biology (Casey 1977; Heinrich 1970; Kingsolver and Woods 1997; Nijhout 1999; Reynolds and Nottingham 1985). They generally have five larval instar stages, with more than 90% of larval growth (mass gain) occurring during the final two instars. Towards the end of the final instar, caterpillars display physiological changes in preparation for pupation (characterized as “wandering” behavior). These pre‐pupal larvae (aka wanderers) burrow into the soil to pupate and then eclose as adult hawkmoths. The animals used in our current study were obtained from an established laboratory colony at the University of North Carolina at Chapel Hill, that has been maintained at UNC since the 1980s (> 250 generations) without the addition of individuals from outside populations (Kingsolver and Nagle 2007). Under laboratory conditions, all life stages are kept at constant 25°C–26°C temperatures with a 14 h light:10 h dark photocycle. Larvae are fed an artificial wheat germ‐based diet (Bell and Joachim 1976), and adults are fed a 10% honey water solution.

### 2.2 Experimental Design

**FIGURE 1:**
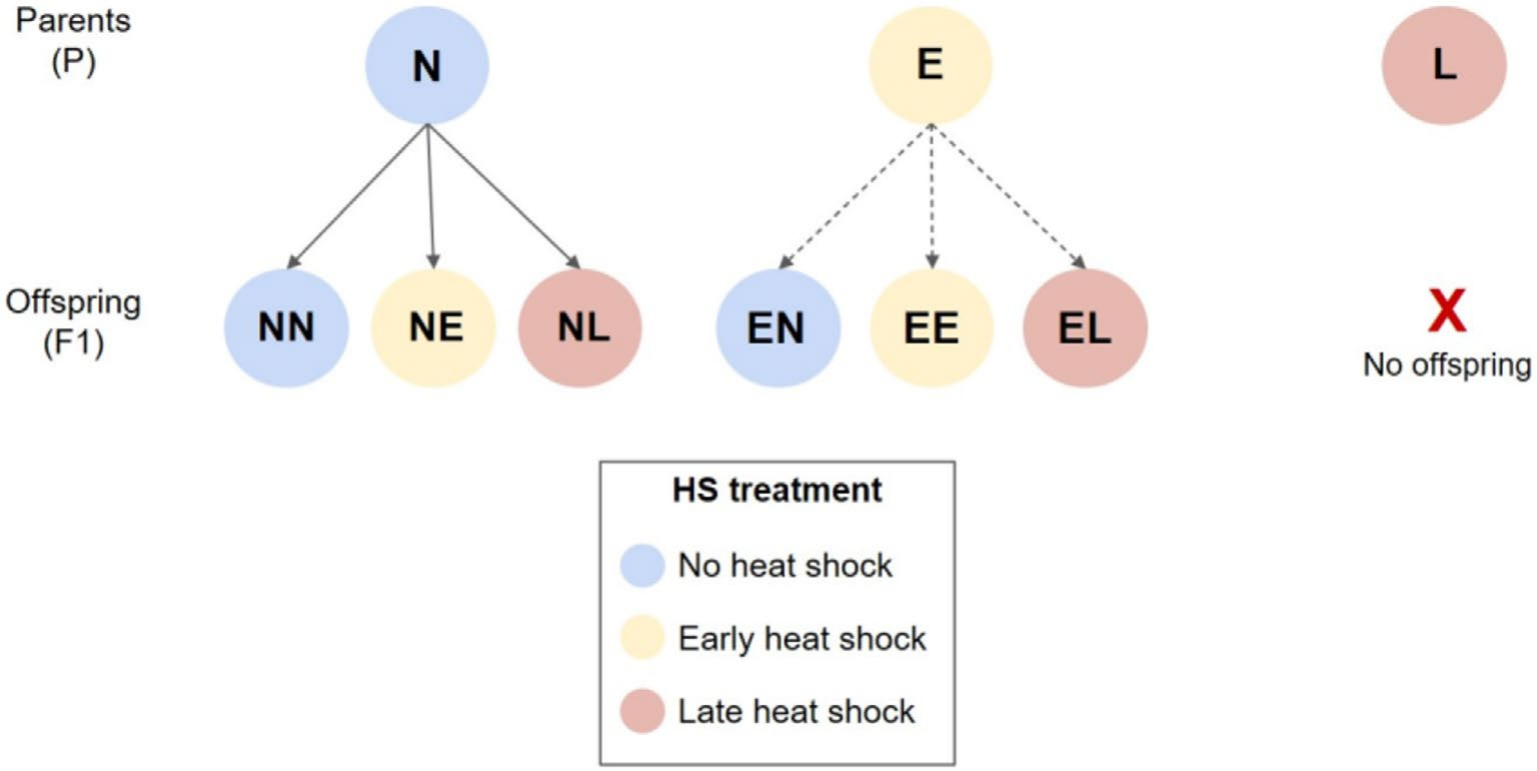
Experimental design of heat shock treatments. Schematic diagram of temperature treatments for parental (P) and offspring (F1) generations. All individuals in both generations were reared in diurnally fluctuating conditions of 25°C ± 10°C and a subset were exposed to either an early (E, 3rd instar, yellow) or a late (L, 5th instar, red) heat shock consisting of 42°C for 24 h one day following molt to instar. Control individuals (N, blue) were not subjected to a heat shock. For the F1 generation, the first letter in the treatment name refers to the thermal treatment that their parents experienced (N, solid arrows or E, dashed arrows). Parents exposed to L heat shocks did not produce any viable offspring, therefore not all possible combinations of parent and offspring treatments were not included.

To determine how the timing of heat stress events during larval development influenced performance and fitness at later life stages, and whether parental thermal exposure affected offspring traits, we performed a factorial experiment across two generations of *M. sexta* (Figure 1). Larvae in both generations were reared in diurnally fluctuating temperature conditions of 25°C ± 10°C. A subset of larvae were exposed to a 24 h heat shock of 42° at either 3rd (early) or 5th (late) instar, resulting in three heat shock (HS) treatments (N = no heat shock, E = early HS, L = late HS). Larvae were reared through to eclosion and adults within the same thermal treatment group were bred in pairs. Eggs produced by the parental generation were used to establish the F1 generation. Originally, we planned on a fully factorial experimental design in which offspring from all three parent HS treatments were divided among the three possible offspring HS treatments. However, no eggs from the late heat shock parent generation hatched (see Results). Therefore, only six offspring treatment combinations (out of the possible nine) were ultimately generated (Figure 1).

Temperature treatments were carried out in environmental chambers (Percival Scientific 36VL) under a 14 h light:10 h dark photocycle. For the 25°C ± 10°C rearing conditions, temperature ramped linearly between 2 h plateaus at the maximum (35°C, 13:00–15:00) and minimum (15°C, 1:00–3:00) over a 24 h period. A separate incubator was set at a constant 42°C for the HS treatment. These experimental thermal conditions were chosen because *M. sexta* larvae grow and develop rapidly with high survival when reared in 25°C ± 10°C conditions (Kingsolver et al. 2015). 42°C is close to the larval upper lethal limits of 44°C–45°C (Casey 1977), therefore, these heat shocks should cause some but not high mortality (Kingsolver et al. 2016). Our goal was to expose larvae to as extreme of a heat shock as possible (while limiting mortality) to maximize our chances of detecting any possible effects.

To establish the first (parent) generation, eggs were obtained from the UNC colony in February of 2020 and maintained at 25°C until hatching. Newly hatched 1st instar larvae were transferred to petri dishes (~10 larvae per dish) with artificial diet and kept at 25°C ± 10°C until molting to 3rd instar larvae. Upon molt to 3rd instar, larvae were assigned to a HS treatment and given a unique ID number. For 3rd instar and all subsequent stages, *M. sexta* were housed individually in dishes. Early heat shock treatment individuals were transferred to 42°C on the day after molt to 3rd instar and then returned to rearing conditions ~24 h later. A similar protocol was followed for late heat shock individuals, but this occurred 1 day following the molt to 5th instar. Petri dishes were checked every 24 h for molting, and food was replenished as needed. Wanderers were transferred to pupation boxes at 25°C and monitored daily to determine the date of pupation. 15 days after pupation, pupae were set up in 16 oz. plastic cups containing a small amount of moist soil and a popsicle stick and covered with a lid until eclosion. Upon eclosion, one male and one female moth from the same HS treatment were paired together. If no mates were available on a given day, moths were placed in a 4°C refrigerator until a suitable mate eclosed (max. of 3 days). The numbers of moths placed in the refrigerator before mating were similar among treatments (Range: 13–14 for P generation; 5–8 for F1 generation). Mate pairs were set up in small, netted enclosures (approximately 30 × 30 × 30 cm), fed with honey water, and provided a tobacco leaf (in water pick) to serve as surface for egg laying. Each mated pair was given a unique ID. These mating enclosures were kept at ~25°C with a 14 L/10D cycle (small night light provided at night). Honey water and leaves were checked regularly and replenished as needed, and eggs were collected from enclosures daily. Larvae that hatched from the eggs of first‐generation moths were set up following the same protocol as above to perpetuate the experiment in the next generation. As much as possible, we ensured relatively equal representation of second‐generation larvae from each first‐generation breeding pair within a treatment group. 24 total families (i.e., breeding pairs) were included in the second generation of our experiment, comprising 12 families from N and 12 from E parental treatments. The median number of offspring per family for N, E, and L direct F1 treatments were 2.5, 3.5, and 2.5 (ranges: 0–13, 0–11, and 1–11), respectively. Four family groups were not represented across all three direct F1 treatments due to producing very few viable eggs that survived to 3rd instar. We ensured that mated moth pairs in the F1 generation were not siblings.

### 2.3 Data Collection

Starting at 3rd instar, survival, age (days), and mass (mg) for each individual were recorded at the start of each larval instar, at wandering, on day 15 of pupation, and on the day of eclosion (measured after wings had fully dried). To quantify change in mass during heat shocks, mass was assessed for all individuals upon entering and then again following both 24 h heat shock events. Sex was determined for each surviving individual at the pupal stage. Fecundity of moths was qualified by collecting and counting the number of eggs laid in each enclosure by the single female. These eggs were then kept at 25°C and checked daily to determine the number that hatched. Adult lifespan was calculated as the number of days from the date of eclosion until the date of adult death if the date of eclosion was the same as the date of pairing in flight cages (i.e., moths kept for one or more nights in a refrigerator at 4°C before being set up in mating cages were not included in adult lifespan calculations). We collected data on the same set of traits in both the parental and F1 generations in order to get a more robust picture of the variation in these measures within each generation; this facilitates our ability to make cross‐generational comparisons. A replication statement for our experimental design is detailed in Table 1. See Table S1 for additional information on sample sizes of individuals and mated pairs by treatment from both generations of the experiment.

**TABLE 1:** Replication statement of the experimental design used to investigate the effects of heat shock on survival to adult, mass and development time of adults, and fecundity of mated moth pairs for both parent (P) and offspring (F1) generations.

	Scale of inference	Scale at which the factor of interest is applied	Number of replicates at the appropriate scale
Parents (P generation)	Individuals	Individuals	Survival: 65, 82, 78 individuals per treatment Mass and development time: 59, 64, 78 adults per treatment
	Mated pairs	Individuals	Fecundity: 18, 20, 18 mated pairs per treatment
Offspring (F1 generation)	Parental mated pairs	Individuals	Survival: 9, 11, 12, 11, 11, 12 parental mated pairs per treatment combination Mass and development time: 9, 11, 12, 11, 11, 11 parental mated pairs per treatment combination
	Parental mated pairs within offspring mated pairs	Individuals	Fecundity: 8, 11, 11, 10, 9, 7 parental mated pairs within offspring mated pair treatments

To determine whether heat shocks affected egg size, a subset of the eggs collected each day from all moth pairs was photographed (along with a calibration slide) using a microscope camera (Omax, 18 MP) attached to a dissecting scope. Egg photos were thresholded to create binary (black and white) images, and egg area (mm^2^) was estimated using Fiji (Schindelin et al. 2012).

### 2.4 Statistical Analyses

All statistical analyses were performed in R (version 4.0.2). Parent (P) and offspring (F1) generations were analyzed separately. Survival to eclosion was assessed using a generalized linear model with a binomial distribution (‘glm’ function from the ‘stats’ package), with HS treatment as a fixed effect for the P generation and HS treatment and parental treatment as interactive fixed effects for the F1 generation. Adult development time, mass at eclosion, and adult lifespan and fecundity measures were modeled using linear mixed effect models (‘lme’ function from the ‘nlme’ package) with HS treatment and sex as fixed effects and individual ID as random intercepts for the P generation. For the F1 generation, HS treatment, parent treatment, and sex were used as fixed effects and individual ID nested within parent's mate pair ID as a random effect. The change in mass during 24 h heat shocks and egg size (area) were also modeled using ‘lme’ as described above, but without the inclusion of sex as a fixed effect. Post hoc pairwise comparisons were conducted with ‘emmeans’ using the Tukey *p*‐value adjustment method.

## 3 Results

### 3.1 Survival

**FIGURE 2:**
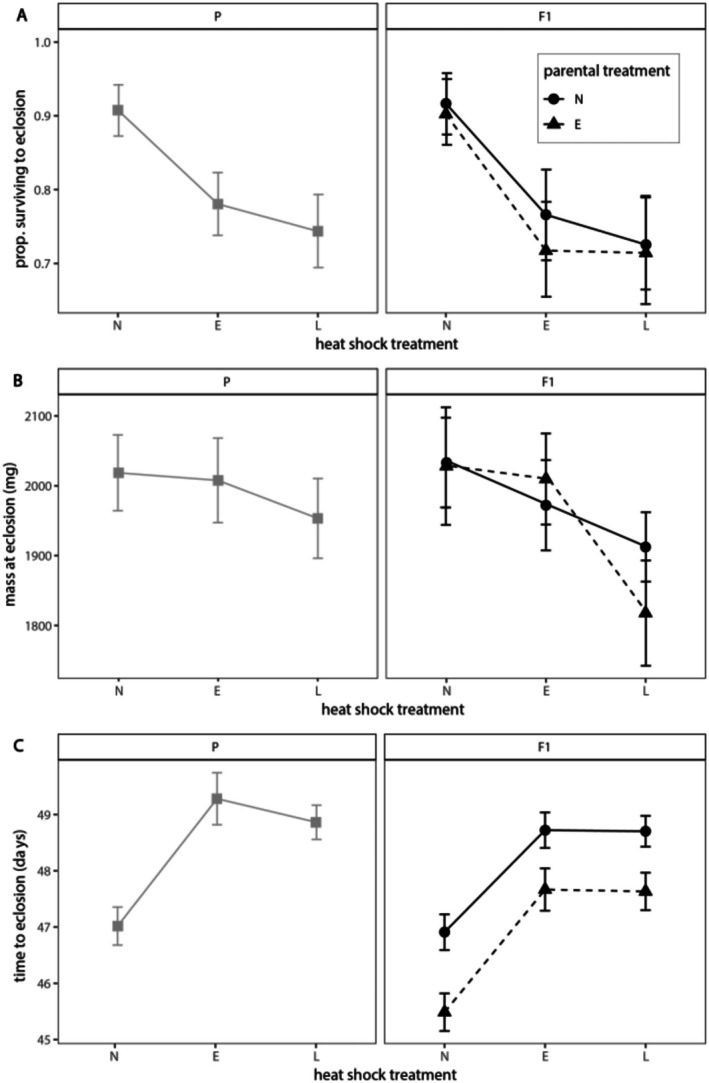
Survival, mass, and development time of *M. sexta* by larval heat shock treatment across two generations. Proportion of individuals that survived to eclosion (A), mean adult mass (mg) at eclosion (B), and development time (days) to eclosion (C) as a function of direct heat shock treatment (N = no heat shock, E = early heat shock, and L = late heat shock). Parental generation (gray, squares) individuals are shown in the left column and F1 generation (black) individuals in the right column. Line types and symbols indicate indirect parental treatment conditions for F1 generation (solid line, circles = no parental heat shock and dashed line, triangles = early parental heat shock). Error bars ± SEM.

Direct exposure to a heat shock (HS) reduced the survival of *Manduca sexta* larvae to eclosion in both P (*χ* ^2^ = −7.24, *p* = 0.0267) and F1 (*χ* ^2^ = −11.53, *p* = 0.0007) generations (Figure 2A, Table S2). Both early and late HS significantly reduced survival compared to non‐heat shock controls (N), and there was no difference in survival based on the timing of the HS (P: *Z* = 0.548, *p* = 0.5838; F1: *Z* = 0.331, *p* = 0.7409). For the F1 generation, indirect effects of parent treatment did not significantly influence offspring larvae survival (Figure 2A, Table S2). While sex could not be determined for individuals that died before pupation, ratios of males to females that survived to adulthood in each treatment group did not differ significantly from a 1:1 expected ratio; therefore, thermal treatments are unlikely to have resulted in differential mortality between sexes (Table S1).

**FIGURE 3:**
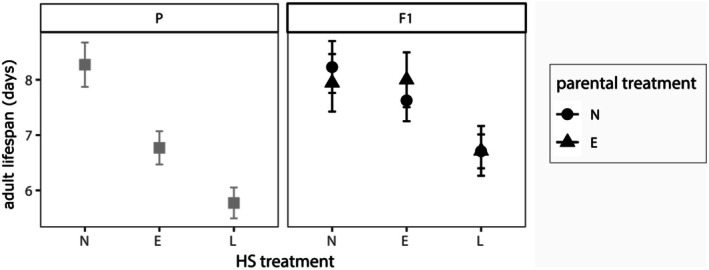
Adult lifespan of *M. sexta* by larval heat shock treatment across two generations. Average number of days from eclosion to moth death for parent (P, gray) and offspring (F1, black) generation by direct HS treatment (N = no heat shock, E = early heat shock, and L = late heat shock). Line types and symbols indicate indirect parental treatment conditions for F1 generation (solid line, circles = no parental heat shock and dashed line, triangles = early parental heat shock). Error bars ± SEM.

Exposure to a heat shock reduced adult lifespan by 1–2 days on average (P: *F*_2,66_ = 14.296, *p* < 0.0001; F1: *F*_2,77_ = 6.075, *p* = 0.0037). This reduction was significant for both early and late heat shocks in the P generation (E: *t*_66_ = −3.429, *p* = 0.003; L: *t*_66_ = −5.164, *p* < 0.0001), but only late heat shocks in the F1 generation (E: *t*_77_ = −1.201, *p* = 0.456; L: *t*_77_ = −2.566, *p* = 0.0329, Figure 3, Table S3). Males tended to have shorter adult lifespans than females on average (P: *F*_1,66_ = 3.16, *p* = 0.0801; F1: *F*_1,77_ = 9.824, *p* = 0.0025). Parental treatment did not indirectly influence adult offspring lifespan (Figure 3, Table S3).

### 3.2 Mass and Development Time

Mass at eclosion was only significantly affected by direct HS treatment for the F1 generation; however, a consistent pattern was seen across both generations (P: *F*_2,177_ = 0.487, *p* = 0.6152; F1: *F*_2,199_ = 5.298, *p* = 0.0058, Figure 2B, Table S3). F1 moths exposed to a late heat shock were significantly smaller on average than controls (*t*_177_ = −2.422, *p* = 0.0432) but only marginally smaller than early heat shock moths (*t*_177_ = −1.811, *p* = 0.169). While not significant, adult mass for late heat shock moths in the P generation did also trend in this same direction, and there was a significant negative effect of late heat shocks for pupal mass for both generations (Figure S1). Consistent with previously reported findings (Grunert et al. 2015), adult males were significantly smaller than females (P: *F*_1,177_ = 64.376, *p* < 0.0001; F1: *F*_1,199_ = 137.248, *p* < 0.0001, Table S3). Indirect effects of parent treatment did not significantly influence offspring mass at eclosion (Figure 2B, Table S3).

Development time, measured as the number of days from hatching to eclosion, was greater by ~1–2 days for both early and late direct heat shock treatments in both generations (P: *F*_2,177_ = 9.93, *p* = 0.0001; F1: *F*_2,199_ = 27.28, *p* < 0.0001, Figure 2C, Table S3). Developmental time showed an across‐generational effect of parental treatment. For F1 individuals produced by early heat‐shocked parents, days to eclosion were reduced across all direct treatments (*F*_1,199_ = 13.75, *p* = 0.0012, Figure 2C). The results presented here focused on the effects of heat shocks for adult mass and development time to eclosion; the effects of heat shocks for other life stages are qualitatively similar and are shown in Figures S1 and S2.

### 3.3 Fecundity

**FIGURE 4:**
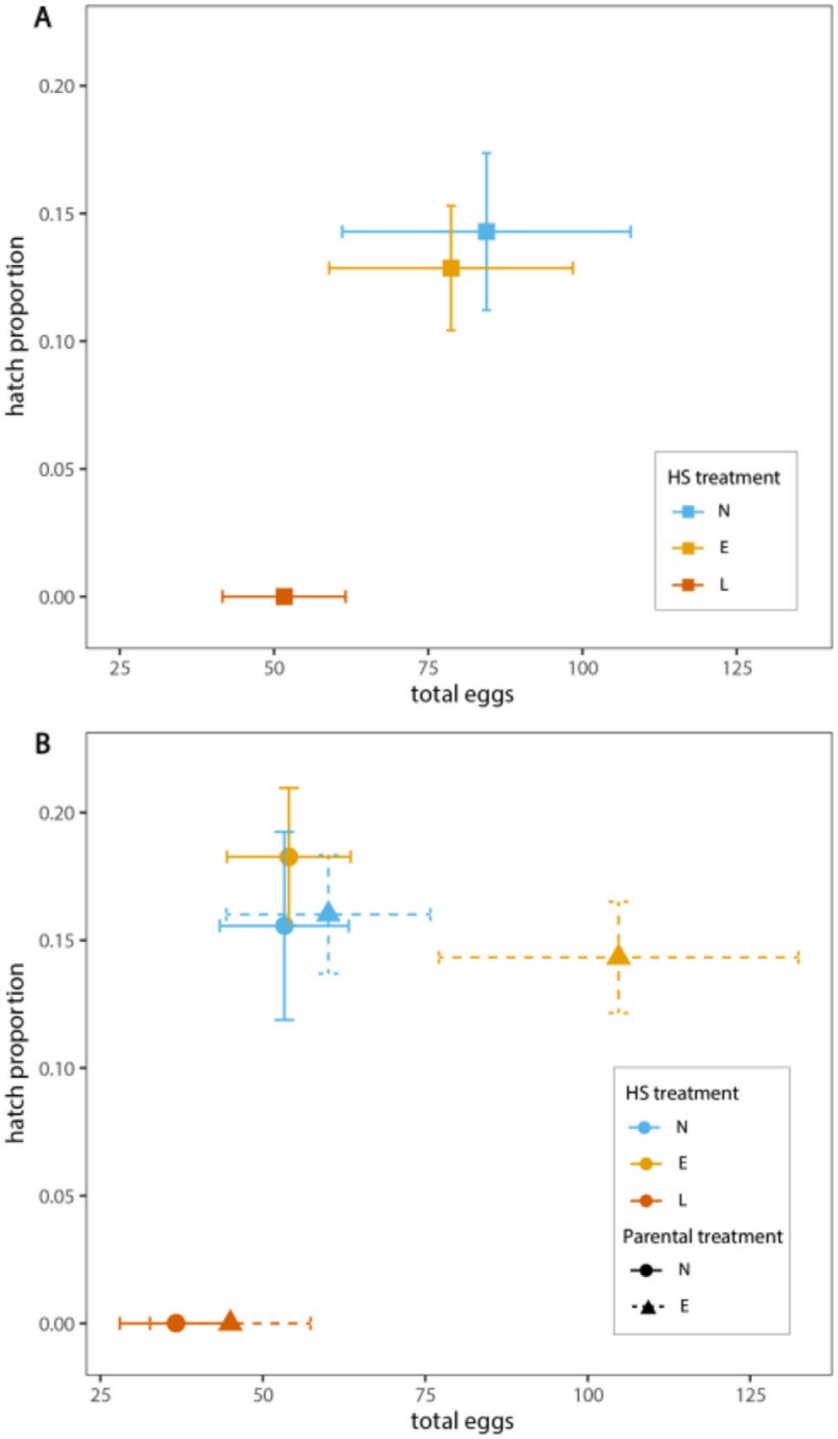
Fecundity of *M. sexta* pairs with heat shock treatments across two generations. The hatch proportion of eggs versus average number of eggs laid for parent (A) and offspring (B) generations of *M. sexta* mated moth pairs. Colors indicate direct heat shock treatment that an individual experienced (blue = no heat shock, yellow = early heat shock, and red = late heat shock). For F1 mate pairs (B), shape and error bar line type indicate indirect parental heat shock treatment, either no heat shock (circles, solid lines) or early heat shock (triangles, dashed lines). Error bars ± SEM.

HS exposure had a strong effect on egg hatching success but not on the number of eggs laid per mated pair (Figure 4). The hatch proportion of early heat‐shocked pairs did not differ significantly from controls, but none of the eggs laid by late heat‐shocked moths hatched in either generation. Although the number of eggs laid per female was not significant for direct HS treatment, late HS pairs trended towards laying fewer eggs (P: *F*_2,53_ = 0.2189, *p* = 0.8042; F1: *F*_2,62_ = 2.9251, *p* = 0.0611). Indirect effects of parent treatment were not significant for total eggs laid or the hatch proportion for the F1 generation (although EE moths were a notable outlier, laying more eggs on average compared to the other treatments).

### 3.4 Egg Size

Most variation in egg size was explained by moth pairs rather than treatment conditions. Direct effects of HS treatment were not significant, although eggs laid by late heat shock moths trended towards being slightly smaller on average compared to early and non‐heat shocked treatments for both generations (Figure S4, P: *F*_2,43_ = 2.58, *p* = 0.0875; F1: *F*_2,41_ = 2.298, *p* = 0.1132). Parental treatment did not indirectly affect egg size for the F1 generation (*F*_1,41_ = 0.201, *p* = 0.6565).

## 4 Discussion

### 4.1 Within‐Generation Effects—Late but Not Early Heat Shocks Significantly Reduce Fertility

Both early and late heat shocks resulted in negative impacts for adult performance and fitness traits, including reduced survival, delayed development to eclosion, and shorter adult lifespans. For most measures, there was not a strong difference in how the timing of heat stress (early versus late) impacted the magnitude of these effects. However, a notable exception to this was for reproduction: moths from late heat shock treatments did not produce any viable offspring (zero egg hatching success), while the fertility of early heat shock moths did not differ significantly from control treatments (Figure 4).

Although less pronounced, late larval heat shocks also had slightly more negative consequences on some other traits. While not always significantly different from early heat shocks, mass at eclosion (and pupal mass), adult lifespan, and numbers of eggs laid per mated moth pair trended towards larger reductions for late heat shock treatments (Figures 3 and 4), though this effect was more subtle than that of egg hatching success. These results offer some support that thermal stress at later developmental stages may be more likely to impact adult fitness, particularly for reproductive success (Knapp and Nedvěd 2013; Ma et al. 2004; Zani et al. 2005; Zhang, Chang, et al. 2015). However, it is likely that critical developmental windows may be more important in explaining this pattern (Burggren and Mueller 2015). In holometabolous insects, the final instar stage (5th in *M. sexta* ) is an important time for mass gain (Reinecke et al. 1980), early reproductive system development (Reinecke et al. 1983), and imaginal disk formation (Rosero et al. 2019). Heat stress during this developmental window (late larval to early pupal stages) may cause greater downstream consequences than thermal exposure before or after this point. One other study found that heat stress at prepupal and early pupal stages in two pyralid moth species reduced fertility by decreasing eupyrene sperm concentrations (Lum 1977). However, heat shocks of later pupae (after most spermatogenesis had occurred) did not cause infertility. While *M. sexta* eggs and pupae show greater thermal sensitivity to high temperatures for development rate and survival compared to larvae (Kingsolver et al. 2011), heat stress of eggs didn't cause lasting impacts for adult traits, including fecundity (Potter et al. 2011). Heat shocks of *M. sexta* pupae can reduce eclosion success and cause morphological perturbations including wing deformities (K. Malinski, *personal communication*), but the downstream consequences of pupal heat stress for adult fertility and fecundity are unknown.

It should be noted that a 24 h heat shock is unrealistic for field conditions; temperatures in the range of 40°–42° are likely to occur for only a few hours during the middle of the day. Because there is still limited understanding about the sensitivity of conditions that can elicit carry‐over and transgenerational effects, particularly for *M. sexta* , we elected to use an extreme 24 h HS for this study to ensure that we did not miss any effects that could be present but less apparent under more natural heatwave conditions. The results of this study can inform future investigations using less severe and/or shorter durations of temperature exposure.

Because we bred males and females from the same treatment together in this experiment, we cannot determine which sex is responsible for the heat‐induced infertility seen in the late heat shock treatment group. Substantial evidence from insects suggests that male reproduction is highly sensitive to thermal stress in many species (Chirault et al. 2015; David et al. 2005; Jørgensen et al. 2006; Porcelli et al. 2017; Sales et al. 2018; Saxena et al. 1992; Zizzari and Ellers 2014), though other studies, including some in Lepidoptera, have found effects of heat stress on female fertility as well (Arbogast 1981; Cui et al. 2008; Green et al. 2019; Janowitz and Fischer 2011). Experiments exploring the reproductive consequences of heat stress to *M. sexta* indicate that both males and female moths may equally contribute to the infertility effect seen in this current study (Alston 2022). One study in a parasitic wasp found that heat shocks may reduce male fertility through injury to developing spermatocytes, leading to reductions in sperm number and/or functional morphological characteristics (Chirault et al. 2015). Lepidoptera produce two sperm types (fertilizing eupyrene and non‐fertilizing apyrene sperm), and both are required for successful fertilization of eggs (Chen et al. 2020; Sahara and Kawamura 2002; Sahara and Takemura 2003) in the Sphingid *Bombyx mori* . Eupyrene spermatocytes in *B. mori* finish meiosis during the final (fifth) larval instar, while the apyrene sperm start meiosis during cocoon formation (Tazima 1964); similar timings for spermatocyte development are likely the case for *M. sexta* . For females, heat shock may cause injury to oocytes and ovarian development, leading to reductions in egg production. We found that the numbers of eggs laid as well as egg size (area) were slightly reduced for heat shock moth pairs (Figure 4 and Figure S4), perhaps indicating that late heat shocks may impact some aspect of oogenesis during female development (although we did not dissect ovarioles of female moths to obtain estimates for total egg production). Overall, the number of eggs laid across treatments in this study was less than those reported in other *M. sexta* mating studies (Ahmad et al. 1989; Madden and Chamberlin 1945; Palumbo and Dahlman 1978). Other confounding factors are likely to influence the rate of female oviposition (e.g., mating success, larval diet, size of mating enclosures, etc.). Life span of female moths may correlate with the numbers of eggs laid; pupal and adult mass is also strongly correlated with egg production (Diamond and Kingsolver 2010).

Surprisingly, even control individuals in this experiment displayed significantly reduced egg hatching success compared to our expectation. Moths from early heat shock and control (no heat shock) treatments produced eggs which had an average hatching success of 10%–20%. In contrast, eggs collected from the UNC lab colony (where larvae are reared at 25°C) typically show hatch percentages closer to 80%–90% (*personal observation*), which is similar to that reported for other *M. sexta* mating studies (Ahmad et al. 1989; Palumbo and Dahlman 1978). This lab colony does exhibit reductions in thermal tolerance and acclimation capacity compared with a NC field population (Kingsolver and Nagle 2007; Kingsolver et al. 2020), potentially as a result of lab adaptation to constant thermal conditions. Therefore, a possible explanation for the low hatching success of control moths in this study is that the 25°C ± 10°C fluctuations experienced across larval rearing contributed to negative fertility effects (Alston 2022). While 25°C ± 10°C rearing conditions were not thought to be stressful for this colony population, performance and fitness had not been evaluated for adult stages prior to this study. Previous studies of lab *M. sexta* indicate that brief exposure to 35°C does not negatively impact larval performance traits, but longer durations of exposure to 35°C can reduce survival and performance (Kingsolver and Nagle 2007; Reynolds and Nottingham 1985). Brief (2 h) exposure to 35°C causes upregulation of multiple *hsp* genes, possibly indicating a stress response; upregulation of these same *hsp* genes occurred even after repeated exposure to this temperature (30°C ± 5°C rearing fluctuations) (Alston et al. 2020). Repeated upregulation of *hsp* and other cellular response pathways may incur a trade‐off in energy allocation (Feder and Hofmann 1999; Folguera et al. 2011). Several studies have documented potential reproductive trade‐offs for insects reared in constant versus variable environments (Cavieres et al. 2020; Folguera et al. 2009).

Both early and late heat shocks caused an increase in the age at eclosion (~1–2 days later than non‐heat shocked controls). Most of this delay can be attributed to a delay to the next stage immediately following the 24 h heat shock (delayed molt to 4th instar for early and delayed wandering for late heat shock treatments) and reductions to development did not continue to accumulate for later stages beyond this initial impact (Figure S2). *M. sexta* larvae halt/reduce consumption during prolonged exposure to temperatures above 40°C, which likely contributes to reduced mass gain for larvae in a heat shock (Figure S3). Molting is triggered once larvae reach a critical weight (Davidowitz and Nijhout 2004), so the time it takes for larvae to make up this mass deficit after recovering from the heat shock is likely a primary driver of this delay.

### 4.2 Across‐Generation Effects—Offspring of Early Heat Shocked Parents Develop Faster

Development time was the only measure that was significantly impacted by the indirect effects of parent heat shock treatment in the next generation. The offspring of early heat‐shocked parents eclosed as adults ~1 day earlier compared to the offspring of non‐heat shocked parents, and this effect was consistent across all three F1 heat shock treatments (Figure 2C). For insect herbivores with multiple generations per year (including *M. sexta* ), shorter development time can increase fitness by two mechanisms: by decreasing larval mortality due to predation and parasitization, and by reducing generation time (Benrey and Denno 1997; Roff 2002). Field studies of *M. sexta* have documented directional selection for shorter larval development time via both of these mechanisms (Eck et al. 2015; Kingsolver et al. 2012). Other studies that test for parental thermal effects tend to focus on adult environment only and have found varying degrees of support for any adaptive potential (Burgess and Marshall 2014; Donelson et al. 2016; Uller et al. 2013). Measures of effects both within and across diverse arrays of taxa don't show clear patterns in responses (Donelson et al. 2018); factors including environmental predictability from one generation to the next, timing of cues during critical windows, and magnitude and/or ramping rates of temperature change could be important and require further investigation (Shama 2017).

Interestingly, faster development associated with indirect effects of E parental treatment did not appear to reduce mass at eclosion (Figure 2B). This suggests that larvae of HS parents were able to eat more and grow faster than larvae of non‐HS parents even when not directly exposed to a heat shock. Whether there are any trade‐offs associated with faster development time is not entirely clear. Survival proportions were slightly (though not significantly) lower for EE individuals compared with the NE treatment group, but overall this trend was not notable for other direct treatment groups (Figure 2A). It is possible that there is some other trade‐off to faster development that was not detected in this study.

Estimates of across‐generation plasticity can be difficult to distinguish from the effects of strong selection within a generation, such as through survivorship bias (Ho and Burggren 2010). While we cannot completely discount this possibility for our current study, we attempted to control for this by splitting siblings in the F1 generation across treatments. Using a laboratory colony is also helpful as there should be less genetic diversity and therefore less variation among phenotypes within treatments.

Because we did not separately test for maternal and paternal effects, we can't speculate much about potential mechanisms. One way for maternal parents to influence offspring traits is through egg provisioning. Egg size did not differ between early and control treatments for our study, but differences in egg provisioning may not necessarily be indicated by larger egg size. For possible epigenetic mechanisms, Gegner et al. (2021) have shown that metamorphosis in *M. sexta* is accompanied by substantial transcriptional reprogramming, facilitated by changes in DNA methylation patterns to key developmental genes including juvenile hormone and ecdysone. This is potentially interesting, given the parental effects in our study resulting in changes to offspring development rates and offers an intriguing avenue for future studies.

While indirect effects of parent treatment were not significant for total eggs laid, EE moth pairs were somewhat of an outlier and laid notably more eggs on average than NE moth pairs. This possibly suggests a biologically relevant, beneficial effect of parental treatment, that is, offspring laid more eggs when they were directly exposed to the same thermal conditions as their parents. However, there was substantial variation in the number of eggs laid by moths within the EE treatment group (Range: 10–345 eggs), and this trend could simply be due to high levels of individual variation among mated moth pairs. A follow‐up study aimed at replicating this effect is needed before arguing this result suggests any evidence of adaptive transgenerational plasticity.

## 5 Conclusion

The effects of temperature on reproduction and other key fitness traits are often primarily considered only for the adult thermal environment. However, as our current study demonstrates, high temperature exposure during earlier life stages can carry over to significantly impact adult traits within a generation as well as offspring traits in the next generation. The timing of temperature exposure is clearly important, and future work should elucidate the boundaries of these thermally sensitive developmental windows and also evaluate the impacts of within‐ and across‐generational temperature effects using additional, more realistic thermal conditions. A more complete understanding of how thermal carry over effects can shape fitness across an organism's lifetime and potentially across subsequent generations will contribute to an improved framework for predicting the impacts of climate change.

## Author Contributions

**Meggan A. Alston:** conceptualization (lead), data curation (lead), formal analysis (lead), investigation (lead), methodology (lead), validation (lead), visualization (lead), writing – original draft (lead), writing – review and editing (equal). **Joel G. Kingsolver:** conceptualization (supporting), formal analysis (supporting), funding acquisition (lead), methodology (supporting), project administration (equal), resources (equal), validation (supporting), writing – review and editing (equal). **Christopher S. Willett:** conceptualization (supporting), formal analysis (supporting), funding acquisition (equal), methodology (supporting), project administration (equal), validation (supporting), writing – review and editing (equal).

## Disclosure

Statement on inclusion: Our study was done on a species that is native to the Southeastern United States, and the laboratory populations used originated from collections from the state of North Carolina, where the researchers were based at the time of the study.

## Conflicts of Interest

The authors declare no conflicts of interest.

## Supporting information


**Appendix S1:** ece372303‐sup‐0001‐AppendixS1.docx.

## Data Availability

Data and code used in this study are available from: https://doi.org/10.5061/dryad.p8cz8w9w6.
