# Supplementary material for: Within‐ and Across‐Generational Effects of Temperature: Exposure of Manduca sexta Larvae to Heat Stress Impacts Future Reproduction and Offspring Development
Source: Ecol Evol. 2025 Oct 12;15(10):e72303. doi: 10.1002/ece3.72303 (PMC12515511; doi:10.1002/ece3.72303)
Supplement: Supplementary file 1 — Appendix S1: ece372303‐sup‐0001‐AppendixS1.docx. [file ECE3-15-e72303-s001.docx]

**Supplemental Information**

| **Treatment** | **Setup** | **Adults** | **Prop. ♀** | **Mated pairs** |
| --- | --- | --- | --- | --- |
| **(A) Parents** | | | | |
| N | 65 | 59 | 0.508 | 18 |
| E | 82 | 64 | 0.484 | 20 |
| L | 78 | 58 | 0.534 | 18 |
| **(B) Offspring** | | | | |
| NN | 36 | 33 | 0.485 | 10 |
| NE | 47 | 36 | 0.444 | 12 |
| NL | 51 | 37 | 0.595 | 12 |
| EN | 41 | 37 | 0.541 | 12 |
| EE | 46 | 33 | 0.515 | 12 |
| EL | 42 | 30 | 0.567 | 10 |

**Table S1 - Sample sizes.** The numbers of individuals by treatment for parent (A) and offspring (B) generations at the start of the experiment (setup), the numbers that survived to eclosion (adults), the proportion of surviving adults that were female (prop. ♀), and the numbers of breeding pairs set up (mated pairs, representing 1 male and 1 female moth from the same treatment).

| **Model** | **AIC** | **df** | **Ln(L)** | **Δdf** | **X^2^** | **p-value** |
| --- | --- | --- | --- | --- | --- | --- |
| **(A) Parents** | | | | | | |
| Full | 221.14 | 3 | -107.57 | -- | -- | -- |
| Drop HS | 224.38 | 1 | -111.19 | 2 | -7.24 | **0.0267** |
| **(B) Offspring** | | | | | | |
| Full | 274.99 | 6 | -131.49 | -- | -- | -- |
| Drop HS | 278.86 | 2 | -137.43 | 4 | -11.53 | **0.0007** |
| Drop P | 269.34 | 3 | -131.67 | 3 | -0.26 | 0.6115 |
| Drop HS:P | 271.08 | 4 | -131.54 | 2 | -0.09 | 0.9560 |

**Table S2 - Survival to ecolsion.** Survival of parent (A) and offspring (B) generations of *Manduca sexta* to eclosion, analyzed using a generalized linear model with a binomial distribution. For (A), the full model included direct heat shock treatment (HS), while (B) included direct heat shock treatment, indirect parental treatment (P), and their interaction as fixed effects. Model comparisons were conducted between the full model and models with the predictor or interaction term of interest removed and compared using Chi-squared tests.

|  | **Adult mass** | | | **Development time** | | | **Adult lifespan** | | |
| --- | --- | --- | --- | --- | --- | --- | --- | --- | --- |
|  | **df** | **F-value** | **p-value** | **df** | **F-value** | **p-value** | **df** | **F-value** | **p-value** |
| (A) Parents | | | | | | | | | |
| intercept | 1 | 4879.949 | **<0.0001** | 1 | 48164.77 | **<0.0001** | 1 | 1376.4404 | **<0.0001** |
| HS | 2 | 0.487 | 0.6152 | 2 | 9.93 | **0.0001** | 2 | 14.2955 | **<0.0001** |
| sex | 1 | 64.376 | **<0.0001** | 1 | 0.01 | 0.9222 | 1 | 3.1599 | 0.0801 |
| (B) Offspring | | | | | | | | | |
| intercept | 1 | 5601.197 | **<0.0001** | 1 | 71282.73 | **<0.0001** | 1 | 1553.1257 | **<0.0001** |
| HS | 2 | 5.298 | **0.0058** | 2 | 27.28 | **<0.0001** | 2 | 6.0750 | **0.0037** |
| P | 1 | 0.512 | 0.4820 | 1 | 13.75 | **0.0012** | 1 | 0.1018 | 0.7532 |
| HS:P | 2 | 0.364 | 0.6957 | 2 | 0.26 | 0.7697 | 2 | 0.4560 | 0.6357 |
| sex | 1 | 137.248 | **<0.0001** | 1 | 1.75 | 0.1872 | 1 | 9.8240 | **0.0025** |

**Table S3** - **Adult mass, development time, and lifespan.** Results of linear mixed model for parent (A) and offspring (B) generations for adult mass, time to eclosion, and adult lifespan. For the parent generation, direct heat shock (HS) and sex were included as fixed effects in the model. For the offspring generation, the model included direct heat shock (HS), indirect parent treatment (P), their interaction, and sex as fixed effects. Individual ID was included as a random intercept.


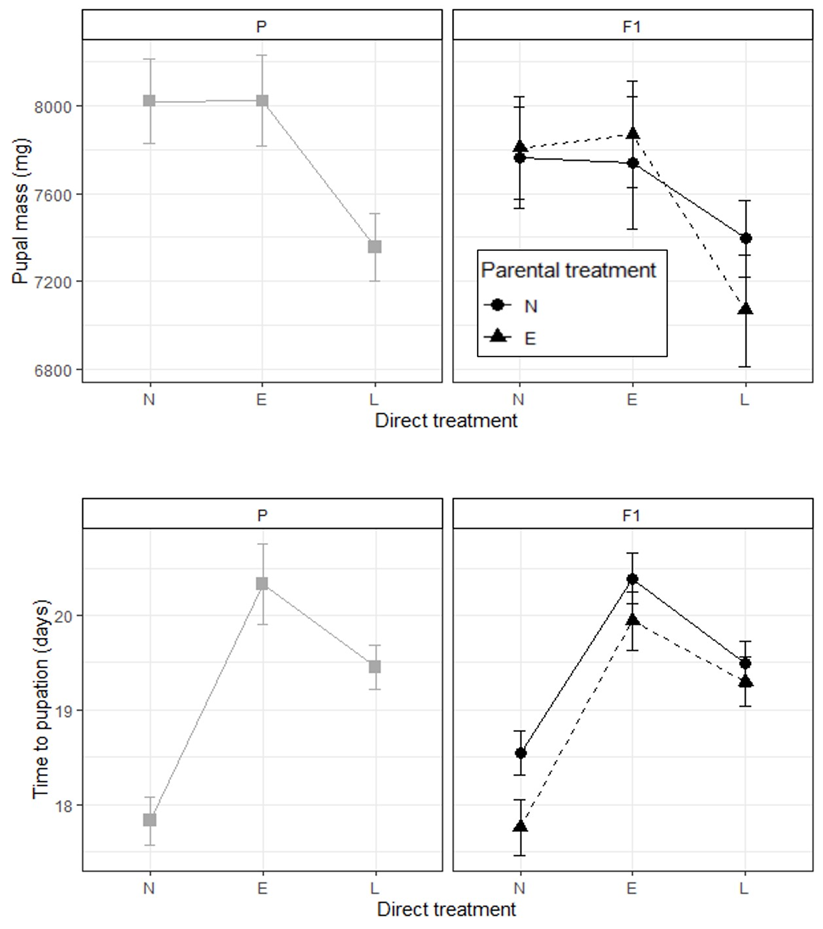


**Figure S1 - Pupal mass and development time.** Mean pupal mass (mg) (top row), and development time (days) to pupation (bottom ow) as a function of direct heat shock treatment (N=no heat shock, E=early heat shock, and L=late heat shock). Parental generation (gray, squares) individuals are shown in the left column and F1 generation (black) individuals in the right column. Line types and symbols indicate indirect parental treatment conditions for F1 generation (solid line, circles=no parental heat shock and dashed line, triangles=early parental heat shock). Error bars ± SEM.


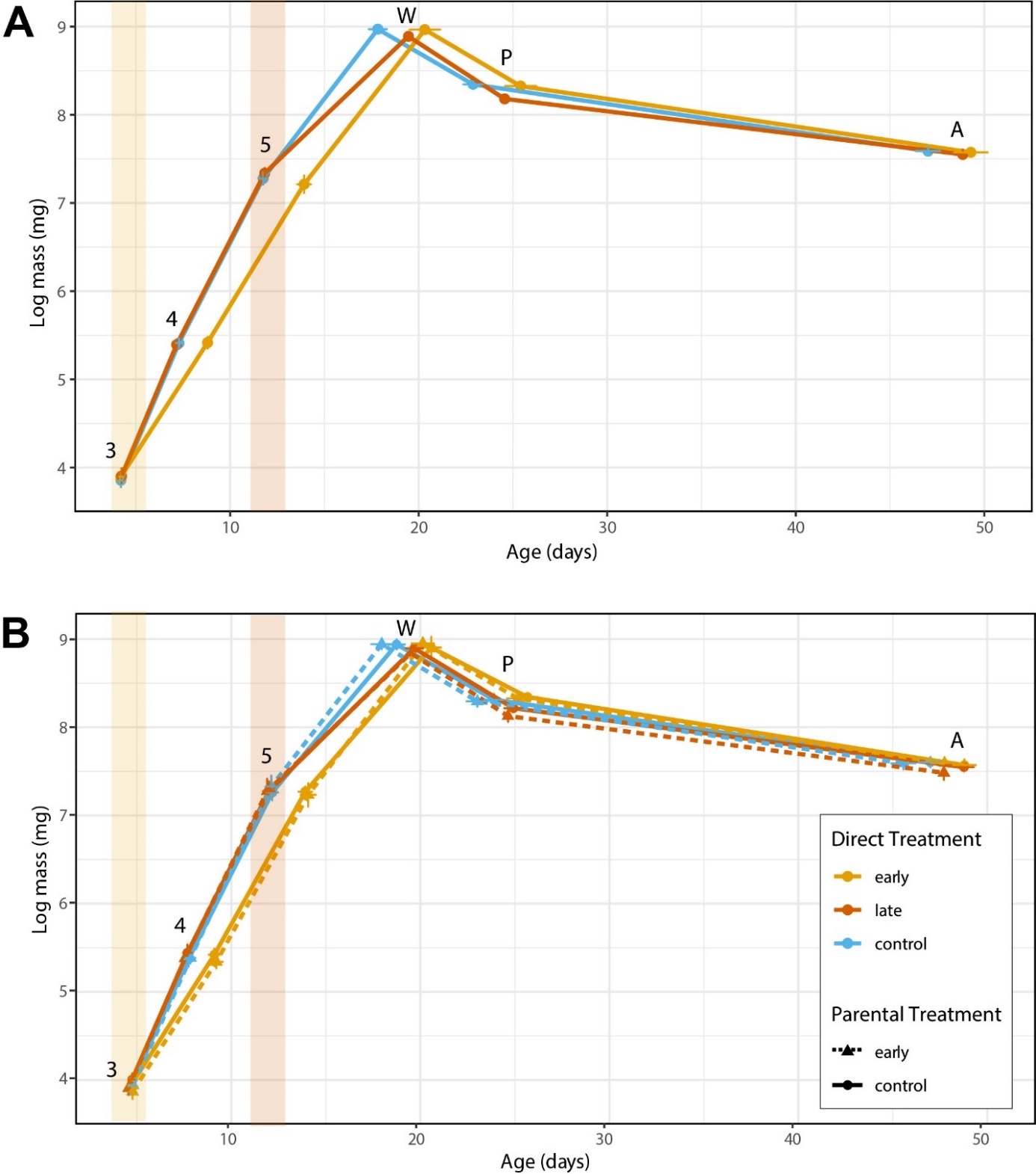


**Figure S2 - Mass and development time across stages.** Log mass (mg) by stage as a function of age (days) to reach that stage for parent (A) and offspring (B) generations (3=3^rd^ instar larva, 4=4^th^ instar larva, 5=5^th^ instar larva, W=wanderer, P=pupa, and A=adult). Colors indicate direct heat shock treatment. Shapes and line type indicate indirect parental treatment for the offspring generation. Error bars ± SEM.


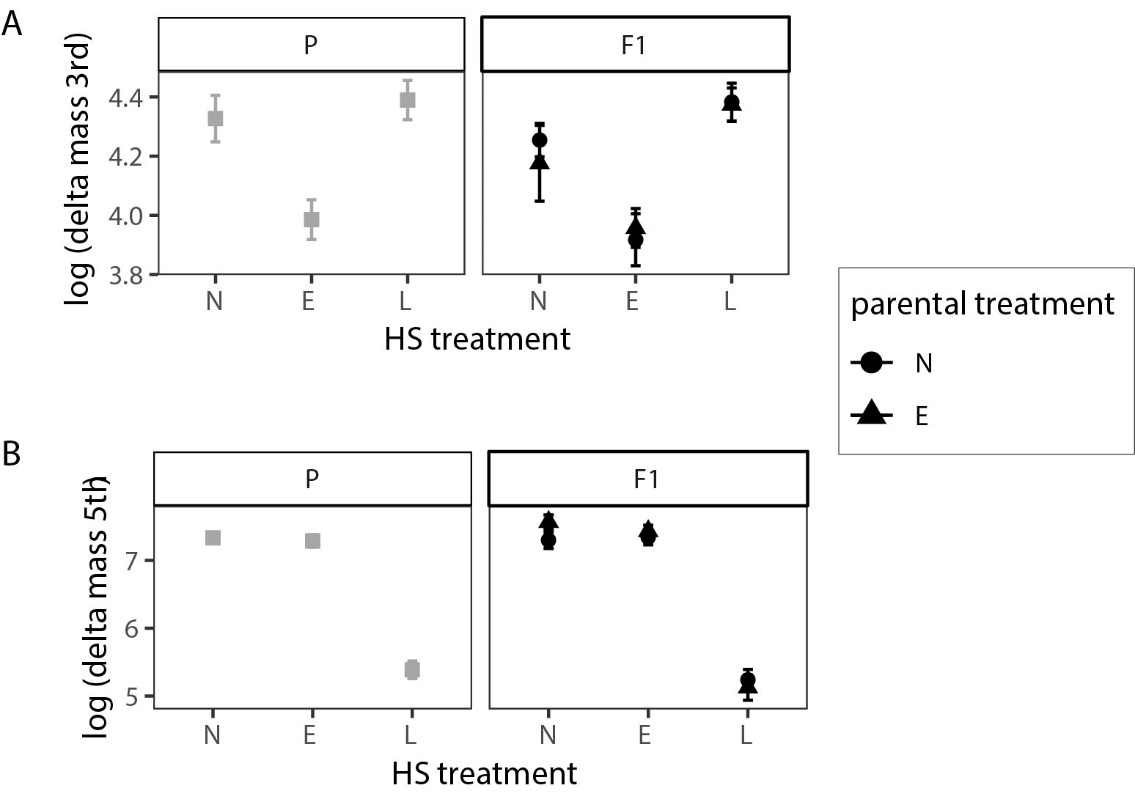


**Figure S3 - Change in mass over 24 h heat shock period.** Log mass differences (mg) calculated as mass at the end of 24 h test period compared to mass at the start of the test period, one day after molt to either 3^rd^ (A) or 5^th^ instar (B), as a function of direct heat shock treatment (N=no heat shock, E=early heat shock, L=late heat shock). Parent generation (P) data are in the left column and offspring generation (F1) are shown in the right column. Shapes indicate indirect parental treatment for the F1 generation. Error bars ± SEM.


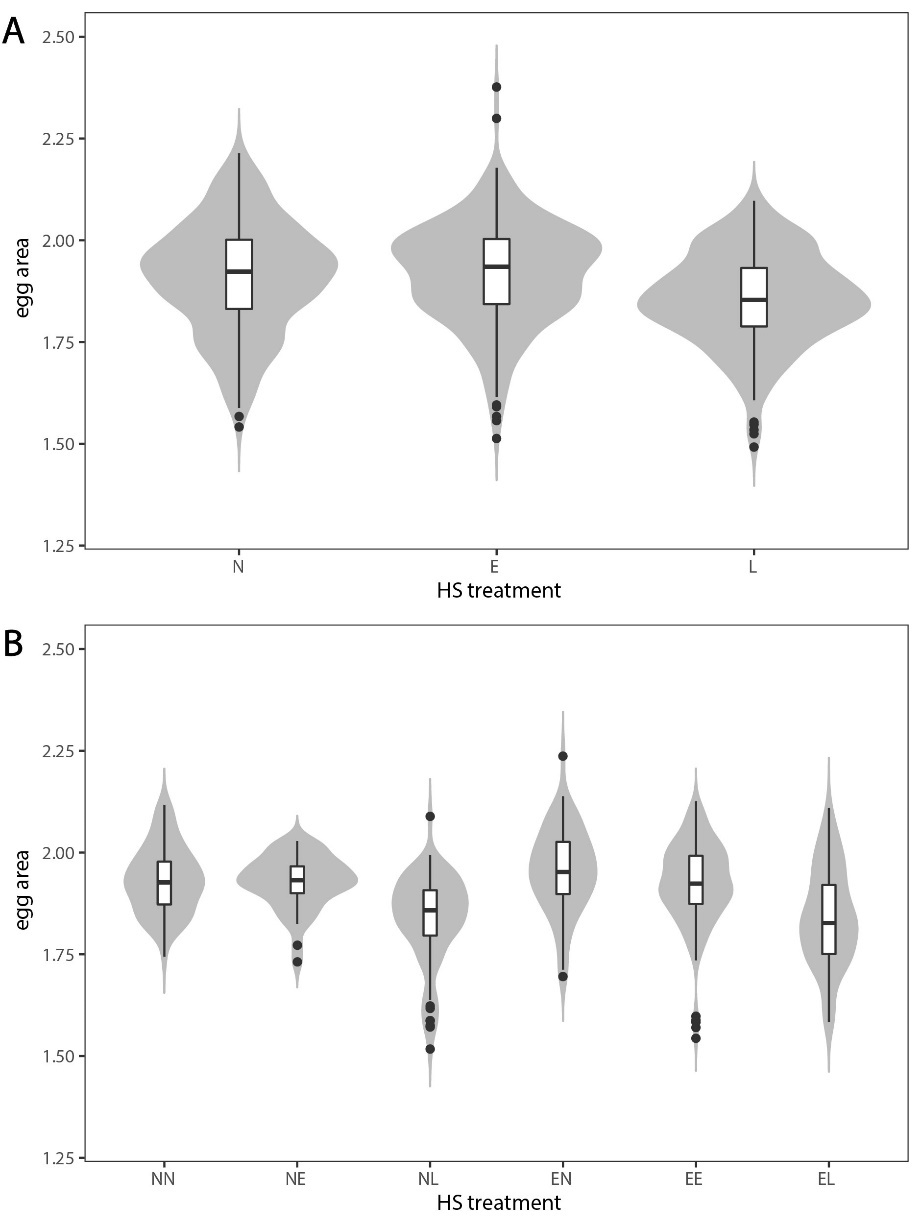


**Figure S4 -** **Egg size.** Violin plots showing egg area (mm^2^) as a function of treatment for parent (A) and offspring (B) generations.
